# Feasibility of Microwave-Based Scissors and Tweezers in Partial Hepatectomy: An Initial Assessment on Canine Model

**DOI:** 10.3389/fsurg.2021.661064

**Published:** 2021-06-17

**Authors:** Khiem Tran Dang, Shigeyuki Naka, Atsushi Yamada, Tohru Tani

**Affiliations:** ^1^Department of Research and Development for Innovative Medical Devices and Systems, Shiga University of Medical Science, Otsu, Japan; ^2^Department of Surgery, University of Medicine and Pharmacy at Ho Chi Minh City, Ho Chi Minh City, Vietnam; ^3^Department of Surgery, Shiga University of Medical Science, Otsu, Japan; ^4^Department of Surgery, Hino Memorial Hospital, Hino, Japan

**Keywords:** microwave surgical device, scissors-type, tweezers-type, clamp crushing, partial hepatectomy

## Abstract

**Purpose:** This study aimed to assess the feasibility of partial hepatectomy (PH) simplified by using microwave-based devices in animal experiments.

**Methods:** PH was performed on 16 beagles using either Acrosurg Scissors (AS) or Acrosurg Tweezers (AT) without hepatic pedicle (HP) control. Parenchymal transection time, Glissonean pedicle (GP) seal time, bleeding volume, bile leak, and burst pressure were recorded. Probable complications were investigated after 4 weeks.

**Results:** Transection time (6.5 [6.0–7.6] vs. 11.8 [10.5–20.2] min, *p* < 0.001) with AT were significantly shorter than with AS. GP sealing times (60 [55–60] vs. 57 [46–91] s, *p* = 0.859) by both devices were nearly similar. Bleeding volume in the AT group was approximately one-fourth of that in the AS group (6.7 [1.4–22] vs. 28.8 [5.8–48] mL, *p* = 0.247). AT created higher burst pressure on the bile duct stumps (*p* = 0.0161). The two devices did not differ significantly in morbidity and mortality after four-week follow-up.

**Conclusion:** Acrosurg devices achieved a safe PH without HP control owing to microwave-based sealing. AS could be used alone in PH, whereas the clamp-crushing function of AT seemed more advantageous in reducing the transection time and blood loss.

## Introduction

Hepatectomy is the main surgical intervention for several liver diseases, especially liver tumors. When performing partial hepatectomy (PH), the most common problems that surgeons encounter are hemorrhage and bile leak (1, 2). Various strategies, including vascular control (Pringle maneuver, total hepatic vascular exclusion, low central venous pressure), and transection techniques (finger fracture, clamp crushing), are used to prevent intraoperative bleeding (3–5). In recent decades, many advanced surgical tools, such as ultrasonically activated devices (UAD), electrothermal bipolar vessel sealers (EBVS), Habib's coagulator, CUSA®, and staplers have also been introduced during liver surgery to reduce the risk associated with PH (6–10). However, none of the latest advanced energy devices could achieve a complete transection of both the hepatic parenchyma and the Glissonean pedicle (GP) safely without the assistance of other surgical tools or hemostatic agents (5, 11–15). The role of such energy devices in the occurrence of post-operative bile leak also remains controversial (16, 17). In practice, the clamp-crush technique under GP clamping is still favored for liver resection at many surgical centers (3, 5, 13, 15, 18), and PH still requires several surgical energy devices so far.

Amid the booming development of modern energy devices, our group invented microwave coagulation surgical instruments (MWCX) for use in many surgical procedures (19–21). The unique microwave dielectric heating by these original MWCX has demonstrated advantages of sealing sizable vessels (20, 22) as well as coagulating fragile parenchyma (spleen, liver) (21, 23). In 2017, two types of microwave surgical devices with improved coagulation power and energy efficiency of MWCX were released commercially: a scissors-type for seamless “seal-and-cut” dissection and a tweezers-type for forceful grasping and sealing of tissues. However, both the old MWCX and the updated scissors and tweezers have never been formally approved for PH as the main surgical instrument. In fact, due to the limitations of currently-used energy devices, hepatectomy needs several surgical tools for dissection, coagulation and cutting of the liver parenchyma as well as the Glissonean pedicle. Our devices could be the solution to this thorny problem.

Based on MWCXs' excellent tissue coagulation reported in previous studies (19–23), we hypothesized that using the new microwave scissors alone would enable PH and GP sealing, whereas the single microwave tweezers would be sufficient to reduce blood loss in this procedure. Therefore, this experimental study was set up to assess the feasibility of using the scissors-type or tweezers-type microwave devices as a single surgical instrument to achieve a safe and simple PH with respect to less bleeding, less risk of bile leaks, and the use of fewer instruments.

## Materials and Methods

### Instruments

**Figure 1:**
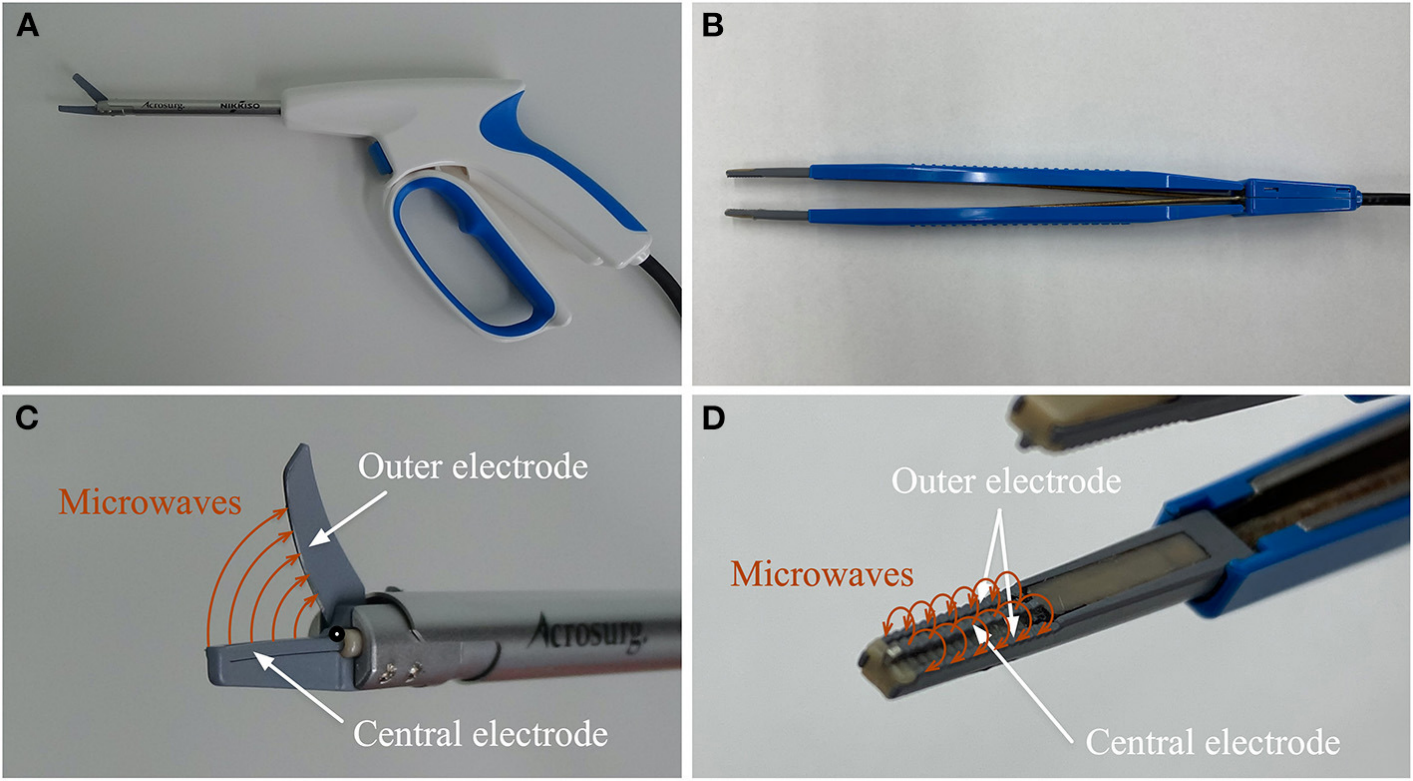
Acrosurg Scissors: the central electrode of the microwave antenna is integrated within the lower blade and the upper blade contains the outer electrode **(A,C)**. Acrosurg Tweezers consists of two branches connected to each other at the hinged end and a bipolar forceps-styled tip. Each serrated jaw includes a central electrode and two parallel outer electrodes of the microwave antenna **(B,D)**.

Two microwave surgical devices (Acrosurg, Nikkiso Co., Ltd., Tokyo, Japan), which have been commercialized based on our MWCX, were used in this study (20, 22, 23). One was the Acrosurg Scissors S09 (AS), and the other was the Acrosurg Tweezers S22 (AT). AS is comprised of a pistol-type handgrip with a microwave emitting button, a 90-mm-long shaft covering a microwave antenna, a 15-mm fixed lower blade, and a 15-mm rotating upper blade (Figure 1A). The thickness of the lower and upper blades is 2.5 and 1.4 mm, respectively. The central and outer electrodes of the microwave antenna are integrated into the fixed and rotating blades, respectively. Thus, microwaves can be emitted from the lower blade to the upper one while performing resection (Figure 1C). In contrast, AT has a pair of fine-serrated jaws whose width and length are 3.2 and 10 mm, respectively, similar to surgical thumb forceps (Figure 1B). Each jaw contains two outer electrodes placed in parallel alongside a central electrode of the microwave antenna so that microwaves circulate around each jaw independently when grasping or clamping tissues (Figure 1D). Both microwave devices are connected to a generator (ASG-01, Nikkiso Co., Ltd., Tokyo, Japan) that produces microwaves at 2,450 MHz (12-cm wavelength).

### Animals

All procedures for the animal experiments were approved by Shiga University of Medical Science (SUMS) Ethical Committee for Animal Research. Sixteen female beagles weighing from 8.5 to 10.5 kg were raised in pathogen-free conditions according to the institutional regulations of SUMS Research Center for Animal Life Science (RCALS). We chose female beagles to facilitate the PH procedure because the male animal has a penis attached alongside the mid-line of the lower half of the abdomen, hindering an extended incision of the abdominal wall. Otherwise, animal sex does not affect the PH short-term outcomes. The beagles underwent general anesthesia with mixed medications: subcutaneous injection of ketamine hydrochloride (Ketalar 500 mg/10 mL, Daiichi Sankyo, Tokyo, Japan) at a dose of 25 mg/kg body weight and medetomidine chloride (Domitor 10 mg/10 mL, Orion Pharma, Espoo, Finland) at a dose of 20 μg/kg body weight. Anesthesia was maintained by inhaled insoflurane 1–2% via an endotracheal tube. The animal were mechanically ventilated with an oxygen-mixed inflow of positive-pressure air to keep the tidal volume in the range of 12–15 mL/kg (24). Physiologic saline 0.9% was intravenously infused at a rate of 180–200 mL/h for intraoperative resuscitation.

### Procedures

**Figure 2:**
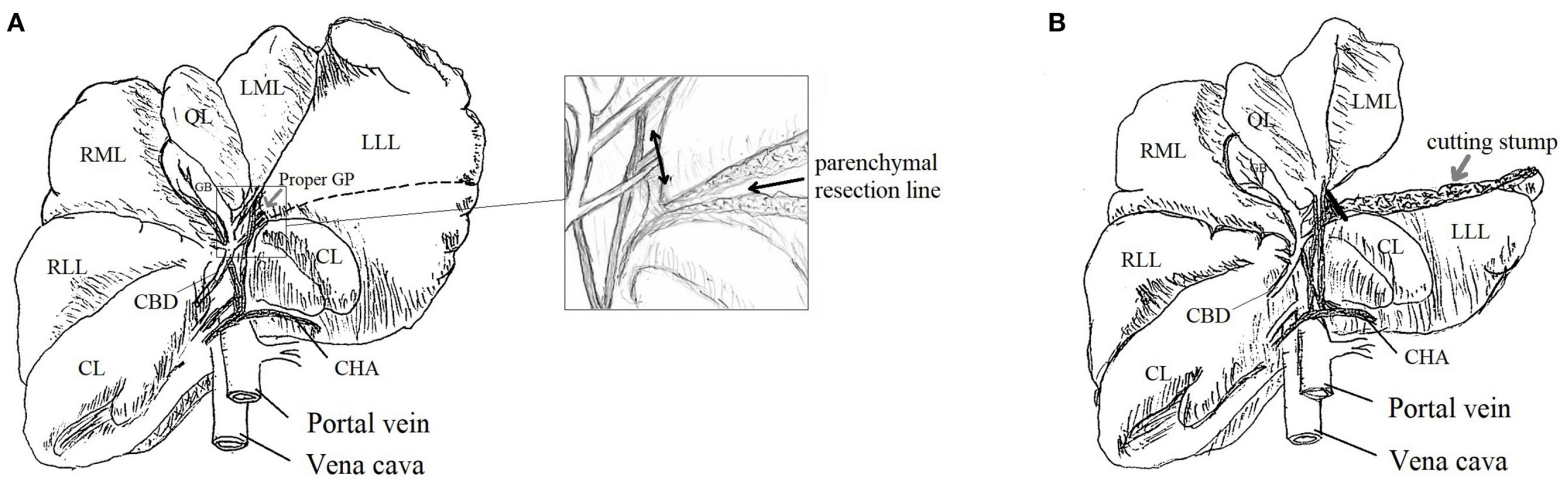
Schema of partial hepatectomy (PH): beagle's liver with demarcated line for parenchymal transection [black dashed line, **(A)**]; parenchymal transection of the inner portion of LLL and its proper GP [double-headed arrow, squared image **(A)**]; liver remnant after PH and the sealed GP stump [black line, **(B)**]. LLL, Left Lateral Lobe; LML, Left Medial Lobe; QL, Quadrate Lobe; RML, Right Medial Lobe; RLL, Right Lateral Lobe; CL, Caudate Lobe; CBD, Common Bile Duct; GB, Gallbladder; CHA, Common Hepatic Artery; GP, Glissonean Pedicle.

All surgical procedures were performed by a single senior surgeon specialized in hepatic surgery (SN) with the assistance of a surgical fellow (KD). Sixteen beagles were allocated to two groups using either AS or AT. After receiving general anesthesia, the beagle was placed in the supine position. A 20-cm-midline incision from the xiphisternum to the umbilicus was made to expose the left lateral lobe (LLL) of the liver. The inner portion of the LLL, which had a separate GP, was selected for PH. The operation included three steps: (1) cut-line marking on the LLL surface; (2) parenchymal transection without hepatic pedicle (HP) occlusion; (3) GP sealing and cutting of the treated portion (Figure 2). The second and third steps depended on which device was employed.

When AS was used, the surgeon performed PH by using only this device. The power output was set at 60 W. The hepatic parenchyma was coagulated and cut seamlessly from the free edge toward the radical end of LLL adjacent to its proper GP. The corresponding GP was, in turn, sealed and cut afterward. When AT was used, the power output was set at 80 W. The hepatic parenchyma was grasped and gently crushed by the AT to isolate crossing vessels and bile ducts inside, while microwaves were concomitantly released from the AT jaws to coagulate the fragmented tissue. The coagulated part was then cut by using Metzenbaum scissors. This cycle was repeated toward the LLL radical end. Similarly, the GP was also sealed by AT and cut with Metzenbaum scissors.

In both protocols, parenchymal transection time and GP seal time were recorded separately. Bleeding volume was estimated in milliliters by subtracting the weight of new gauzes from the blood-soaked gauzes after the surgery using a conversion rate of 1 g = 1 mL (25). If excessive bleeding occurred, the lost blood was to be harvested by a suction system and its volume added to the total volume. The length and thickness of the resected portions and GP diameter were measured.

Before abdominal closure, new gauze was applied on the cutting stump of the remnant liver to check the bile-leak coloration; leaking was confirmed if yellowish fluid tinged the gauze. The dog was then transferred back to RCALS for post-operative follow-up. After 4 weeks, re-laparotomy was conducted to confirm any chronic adverse events (bleeding, abscess, ascites, adhesion). The extrahepatic bile ducts (EBDs) were harvested to test *ex vivo* the EBD sealing. In this test, a small catheter was inserted into the open lumen of the EBD. Physiologic saline 0.9% was administered gradually by an electric pump (KDJ20, KD Scientific, Inc., Holliston, Massachusetts) connected to a pressure amplifier (PA-001, Star Medical, Inc., Tokyo, Japan). Burst pressure was defined as the highest intraluminal pressure prior to EBD stump leakage (16).

All resected portions containing the GP stump and sealed EBDs were fixed in 10% neutral-buffered formalin, paraffinized, and sectioned into 2-μm-thick slides for hematoxylin and eosin (H&E) staining. Histological features were evaluated under a light microscope (Nikon Eclipse 90i, Nikon Corp., Tokyo, Japan) with image processing software (Image-Pro Plus version 7.0J, Media Cybernetics, Inc., Bethesda, Maryland).

### Statistical Analyses

The data are presented as medians with interquartile ranges for continuous variables and as actual numbers for categorical variables. The cutting surface of the liver was approximated by an ellipsoidal shape. Thus, the transection area *S* (cm^2^) and transection speed *v* (cm^2^/min) were calculated by the following equations:

$$\begin{array}{l} S=\pi ab\;(a\;and\;b\text{ represent half of the section length}\\ \text{ and thickness})\\ \;v=S/t\;(t\text{ represents the transection time}) \end{array}$$

The bleeding rate (mL/cm^2^) was also calculated by dividing the bleeding volume by the transection area. All data were analyzed by a statistical software package (Stata 12.0, StataCorp., Lakeway Drive, Texas). Fisher's exact test was applied to compare two categorical variables, whereas the Wilcoxon rank-sum test was applied to test the difference between two quantitative groups; *p* < 0.05 was considered significant.

## Results

The PH outcomes using the Acrosurg devices (AS and AT) are presented in Table 1. The transection areas by both devices were not significantly different (*p* = 0.082). AT required only half of the transection time of AS to finish the parenchymal dissection (median: 6.5 vs. 11.8 min, *p* < 0.001). As a result, the transection speed of AT was two-fold higher than that of AS (median: 2.1 vs. 0.9 cm^2^/min, *p* < 0.001). AT sealed GP that had a larger diameter (median: 11.1 vs. 7.9 mm, *p* = 0.003) within a similar seal time (median: 60 vs. 57 s, *p* = 0.859) compared to AS.

**Table 1:** Outcomes of partial hepatectomy using Acrosurg Scissors (AS) and Tweezers (AT).

**Outcome**	**AS**	**AT**	p^*^
Total operated cases	8	8	
**Parenchymal transection**			
Transection area (cm^2^)	12 [11.2–12.4]	12.7 [12.2–15.9]	0.082
Transection time (min)	11.8 [10.5–20.2]	6.5 [6.0–7.6]	0.0008
Transection speed (cm^2^/min)	0.9 [0.7–1.2]	2.1 [1.9–2.2]	0.0007
**GP sealing**			
GP diameter (mm)	7.9 [7.7–8.9]	11.1 [10–12.8]	0.003
GP seal time (s)	57 [46–91]	60 [55–60]	0.859
**Intraoperative bleeding**			
Bleeding volume (mL)	28.8 [5.8–48]	6.7 [1.4–22]	0.247
Bleeding rate (mL/cm^2^)^§^	2.4 [0.5–3.5]	0.5 [0.1–1.7]	0.292
**Bile leak after hepatectomy**	0	0	1.0
**After 4-week follow-up**			
Total cases observed	8	8	
Adhesion	6	3	0.351
Complication	1 (Ascites)	1 (Bile leak)	1.0
Death	1 (Gallbladder bed bleeding)	0	1.0

* *p-value calculated by the Wilcoxon rank-sum test for quantitative data represented as median [interquartile range]; and by Fisher's exact test for qualitative data*.

§ *Bleeding rate calculated from the bleeding volume and transection area*.

*GP, Glissonean pedicle*.

The bleeding volume in the AT group was estimated at less than one-fourth of that in the AS group (median: 6.7 vs. 28.8 mL, *p* = 0.247). PH using AS often encountered more oozing of blood than with AT (median bleeding rate: 2.4 vs. 0.5 mL/cm^2^). Most blood loss collected during the operation were determined to be parenchymal transection-related bleeding because all GP sealing was accomplished by both devices without any considerable bleeding. No blood loss was harvested by using a suction system. There was no bile leak at the cutting surface immediately after PH in both groups.

All dogs in the AT group were healthy after 4 weeks, whereas one dog from the AS group died on the 2nd post-operative day. Autopsy revealed many clots had accumulated at the infrahepatic recess, surrounding the gallbladder bed. The gallbladder of this dog had been removed because of a gallbladder laceration when performing PH. The old cutting stump remained dry without clotting or ongoing bleeding. There were no differences regarding complications between the two groups (*p* = 1.0). A pale bile-tinged coloration of the cutting stump was discovered in one case in the AT group that implied a probable bile leak. Adhesions were observed more frequently when using AS than with AT (*p* = 0.351).

*Ex vivo* experimental data are shown in Table 2. Although the EBD stumps sealed by both devices exhibited excellent burst pressure, AT-induced seals withstood a significantly higher burst pressure than that of AS-induced seals (median: 806.5 vs. 607.5 mmHg, *p* = 0.0161), and AT could seal larger EBDs (*p* = 0.0045).

**Table 2:** Extrahepatic bile duct (EBD) sealing by two acrosurg devices.

**Outcome**	**AS**	**AT**	p^*^
Number of trials	6	6	
Diameter (mm)	1.8 [1.8–1.8]	3.4 [2.8–3.4]	0.0045
Burst pressure (mmHg)	607.5 [472–705]	806.5 [776–889]	0.0161

* *p-value from Wilcoxon's ranksum test, data represented as median [interquartile range]*.

*AS, Acrosurg Scissors; AT, Acrosurg Tweezers*.

**Figure 3:**
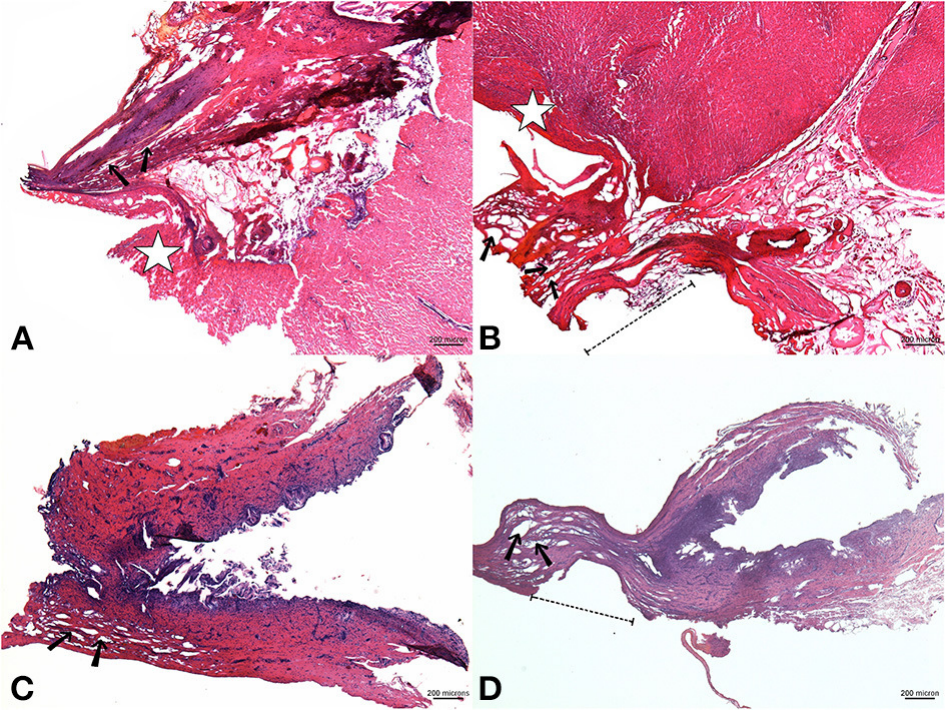
Upper images: a microscopic view of a sealed Glissonean pedicle (GP) by AS **(A)** and AT **(B)**; Lower images: sealed EBD using AS **(C)** and AT **(D)**. The GP stumps in both groups were fused; their structures were histologically indistinct (H&E stain, x40). The vicinity of the GP was covered by a layer of deformed hepatocytes, which lost their normal texture [star mark, **(A,B)**]. Vacuolization caused by microwave energy existed along the GP's interstitial zones [black arrow, **(A,B)**]. Vacuole-like spaces also appeared in the EBD stumps [black arrow, **(C,D)**, x40] whose sealed edges revealed a deformed segment that fitted the serrated-jaws of the tweezers-type device [dashed lines, **(B,D)**]. AS, Acrosurg Scissors; AT, Acrosurg Tweezers; EBD, Extrahepatic bile duct; H&E, Hematoxylin and Eosin.

Microscopic examination revealed that the GP stumps sealed by both devices showed complete fusion of the pedicle as well as its inside components, which were histologically indistinguishable. The surrounding area was covered by a layer of destroyed hepatocytes (Figure 3A). It was possible to observe the vacuolization in the interstitial zone alongside the GP, which indicated the unique characteristic of microwave coagulation in the tubal structures. The EBD samples revealed similar pathological features: all layers were denatured and fixed with interlayer vacuole-like spaces that created total occlusion of the sealed edges (Figure 3C). In particular, there was a flattened, well-coagulated segment in specimens sealed by AT due to the forceful compression from the serrated jaws of the tweezers (dashed lines, Figures 3B,D).

## Discussion

In this study, we assessed the contribution of two Acrosurg devices separately to achieve safe and simple PH. To date, one of the most widely-accepted strategies to control blood loss in PH is the combination of inflow occlusion (Pringle maneuver) with some energy devices (EBVS, UAD) or a dissector (CUSA®, water jet) (7, 8, 18, 26, 27). As a result, a PH procedure requires several surgical instruments (dissectors, energy-based sealers, stapler etc.). Acrosurg devices have already proven their sealing capability in previous studies (19–21, 23). Hence, it was reasonable to test AS and AT as new instruments to reduce the number of instruments used for a PH.

In the present study, both AS and AT could achieve a complete parenchymal transection with minor bleeding (<30 mL). Even when adding up to 25% of the estimated blood loss, which was considered the “concealed” bleeding during the operation (25), to the total volume, the subsequent amount of bleeding remained <50 mL. The bleeding rates when using AS or AT were lower than those reported by recent studies that employed conventional energy devices in open hepatectomy. Although it is difficult to compare experimental data with clinical outcomes, the use of AT resulted in a bleeding rate that was less than one-sixth of the published clinical data (0.5 vs. 6.6, 5.04, 3.19, or 3.4 mL/cm^2^) (7, 8, 15, 28). Both AS and AT gave positive outcomes in terms of transection speed. Although the speed with AS seemed equivalent to other energy devices, AT outperformed energy devices applied in previous studies (2.1 vs. 1.07, 1.11, and 1.16 cm^2^/min) (7, 8, 28). The overwhelming cutting speed and minor bleeding associated with the use of AT compared to other energy devices (even with AS) might be explained by its sealing process. AT has serrated jaws to grasp and crush the tissue, which is then flattened under forceful compression. Owing to the crushing of the 3.2-mm-wide jaws, intrahepatic structures (artery, portal vein, bile duct) were skeletonized within a narrow strip (equal to 3.2 mm) that allowed the surgeon to observe them clearly before conducting the coagulation. The use of AS precluded the identification of the underlying structures that were sandwiched by the hepatic parenchyma during the seamless transection. Therefore, the sealing zone could sometimes undergo premature resection that caused the oozing of blood on the cutting surface, requiring more time for recoagulation during the transection phase. Clinically, AS is suitable to perform a simple hepatectomy such as wedge resection whereas AT could be used for large liver resections which need firm hemostases. It is also possible to combine AT and AS to shorten the transection time.

The good results of Acrosurg devices are also attributed to the heating mechanism of microwaves. Microwaves are able to penetrate to the core of the induced liver portion and enable homogenous coagulation of the targeted tissue from inside to outside (29). Their dielectric heating also engenders less thermal injury, avoiding undesirable destruction of collateral structures (21, 23). The microwave sealing process, combined with the scissors-like design, helped AS achieve PH without instrument exchanges. If such excellent sealing is accompanied by the use of the clamp-crushing AT, a surgeon only needs a pair of Metzenbaum scissors to perform nearly bloodless PH within a short transection period. In practice, there are some energy devices, such as EBVS or UAD, that are available for parenchymal dissection, but neither has been applied as the sole instrument for the whole procedure of PH (7, 8, 18). Hence, using Acrosurg devices might be likely to simplify the PH procedure.

Besides parenchymal dissection, the GP, which consists of portal triads, is often ligated separately by tying knots, placing sutures, or using staplers in a conventional PH (5, 9, 10, 13, 18). In contrast, Acrosurg alone could seal the GP as well as EBDs flawlessly. In the experimental condition, our study reported the creation of an EBD stump capable of withstanding significantly higher burst pressure compared to other EBVS/UAD instruments reported in a previous study (16). This finding was consolidated by histological examination where the GP and EBD stumps, deemed to be single large vessels, were shown to be definitely fused and fixed, demonstrating seal integrity. During this experiment, regardless of whether AS or AT was used, the large GP was slowly sealed and cut sequentially in two or three overlapping cycles with only a less-than-5-mm, intervening GP segment to ensure an adequate seal. These results showed that Acrosurg devices were able to seal these structures perfectly. Furthermore, the results with EBDs demonstrated that Acrosurg devices could secure the coagulation of smaller intrahepatic bile ducts, which showed no cholorrhea immediately after the operation.

The 4-week follow-up also revealed positive endpoints. The dead beagle in the AS group was attributed to intra-abdominal hemorrhage due to excessive bleeding from the gallbladder bed. This case was considered a PH complication, but it was not directly related to the bleeding from the parenchyma or GP stump. This indicated that all stumps had been completely sealed. Nevertheless, a bile leak was still detected with the naked-eye in one case in the AT group. To eliminate the drawback of macroscopic assessment, we recommend that bilirubin tests of ascites or abdominal fluid should be carried out in both the acute and chronic post-operative stages.

In this study, we did not perform a comparative experiment between our devices and other conventional energy devices (Harmonic, LigaSure or CUSA®) because there was no one currently available device that could be used to perform the entire PH procedure by itself. Also, the function of Acrosurg for hepatectomy has not been presented earlier. Our work is the first study to prove that PH and Glissonean pedicle dissection could be achieved safely and simply with a single microwave device in an experimental model. We used a number of 16 beagles to comply with the principles of 3Rs in animal experiments (implemented by SUMS) but still ensure the statistical significance of the research. These preclinical data are the essential evidence to introduce Acrosurg to PHs on humans.

The primary limitation of this study was the lack of intraoperative monitoring of blood pressure and central venous pressure whose fluctuations might affect bleeding during liver resection (14). This study was performed on a normal liver, whereas performing PH on a cirrhotic liver is not an exceptional circumstance (30). Therefore, functional assessment of Acrosurg on abnormal liver texture should be conducted, especially focusing on the potential of a non-bleeding parenchymal dissection without prior HP control. A safe PH without inflow occlusions helps patients avoid the risk of liver function impairment as well as hemodynamic disturbance, both life-threatening events in cirrhotic or chemotherapy-induced livers.

In conclusion, two microwave-based Acrosurg devices were able to achieve a complete parenchymal transection as well as a GP seal without prior HP occlusion in an experimental model. AS could be employed as a sole instrument to perform PH whereas AT, which combined a clamp-crushing maneuver with microwave sealing, demonstrated a quasi-bloodless PH in a shorter operation time than the seamless transection achieved using AS. Both devices allow surgeons to perform a safer PH with very few instruments. The results of this study indicate that these Acrosurg devices could be tested for PH in clinical trials.

## Data Availability Statement

The raw data supporting the conclusions of this article will be made available by the authors, without undue reservation.

## Ethics Statement

The animal study was reviewed and approved by Shiga University of Medical Science (SUMS) Ethical Committee for Animal Research.

## Author Contributions

KD: participated in study design, performed the experiment, collected and analyzed the data, and wrote the manuscript. SN: designed the study, performed the experiment, provided critical comments, and revised the manuscript. AY: participated in the experiment and data collection, provided critical comments, and edited the manuscript. TT: designed the study, performed the experiment, critically revised the manuscript, and approved the submission. All authors contributed to the article and approved the submitted version.

## Conflict of Interest

TT was the inventor of the microwave surgical devices (MWCX), and his company received royalties provided by intellectual property of MWCX. The remaining authors declare that the research was conducted in the absence of any commercial or financial relationships that could be construed as a potential conflict of interest.
